# A holistic approach to addressing the degree awarding gap: a perspective

**DOI:** 10.1042/ETLS20253031

**Published:** 2026-03-05

**Authors:** Prachi Stafford

**Affiliations:** Senior Lecturer in Biological Sciences, EDI (Equality Diversity and Inlcusion) Lead, School of Biosciences and Chemistry, Sheffield Hallam University, Sheffield, S1 1WB, U.K.

**Keywords:** belonging, degree awarding gap, hidden curriculum, inclusion, representation, teaching and assessment

## Abstract

The degree awarding gap (DAG), disparities in degree outcomes across student demographics, remains a persistent challenge in UK higher education. At Sheffield Hallam University (SHU), the School of Biosciences and Chemistry has implemented a series of inclusive initiatives to address this issue across the student journey.

Drawing on culturally responsive pedagogies, the work targets belonging, representation and the hidden curriculum through outreach, peer-led networks, visible role models, inclusive seminars and embedded equality, diversity and inclusion (EDI). Mini case studies highlight collaborative efforts to diversify curricula, co-create assessment rubrics, support identity-affirming student groups and integrate EDI into school operations.

These initiatives have enhanced student engagement, staff confidence and the visibility of underrepresented voices. This article calls for sustained, school/institution-wide commitment to equity, recognising that closing the DAG requires long-term structural change, shared responsibility and a culture where all students feel they belong and can succeed.

## Introduction

The term degree awarding gap (DAG) refers to persistent disparities in degree outcomes between students from different backgrounds, shaped by a complex interplay of structural, institutional and individual factors [1]. DAG, with regards to ethnicity, first entered United Kingdom higher education (HE) discourse via the NUS *Race for Equality* report [2] and gained momentum with the Runnymede Trust’s *Aiming Higher* [3]. Today, DAG is recognised globally as a systemic issue reflecting the intersectionality of student identities, including race, gender, socio-economic status and disability, collectively affecting students’ educational experience. For example, in the UK, the Office for Students (OfS) reports graduates from a Black ethnic background were 15.0 percentage points less likely to receive a first-class degree than the all-ethnicities average in 2022–23 [4].

Despite years of targeted interventions, progress in closing the DAG remains slow [5]. This complex challenge [6,7] requires more than siloed, reactive responses; it demands a holistic, inclusive approach embedded across the student journey. To guide our initiatives within the school, we adopted the Hubbard and Gawthorpe Inclusive Education Framework [8], which identifies interconnected domains: Structures and Processes, Curriculum Design and Delivery, Assessment and Feedback, Community and Belonging, and Pathways to Success, each rooted in equity, inclusion and belonging. Our initiatives were mapped to these domains to ensure strategic alignment and impact.

This piece showcases initiatives I have led or supported as Equality, Diversity and Inclusion (EDI) lead within the School of Biosciences and Chemistry at Sheffield Hallam University. Through a collaborative whole-school approach, we have successfully embedded inclusive practice across the School’s community, teaching, learning and assessment which are summarised below.

## Mini case studies

### Structure and processes

#### Embedding EDI in institutional culture

*Rationale*: Strategic integration of EDI into governance and operation is essential for meaningful and sustained change [9].

*Initiatives:* EDI became a standing item in School meetings, repositioning it as a core strategic priority and aligning with the Advance HE Equality Framework [9]. Staff integrate EDI into teaching, policy and planning [10]. Visibility and belonging were promoted through pronoun use in email signatures and distributing pronoun pins, reinforcing authenticity and allyship via Inclusive Leadership behaviours.

*Impact*: Inclusive practices became embedded with staff engagement in EDI, fostering a visible culture of belonging across the School. Module lead: *Student feedback highlighted appreciation for pronoun pins and inclusive email signatures, as signals of a safe space and a cultural shift towards normalising inclusion*.

#### Inclusive timetabling

*Rationale*: Timetables can unintentionally disadvantage students with cultural or religious commitments. Our observations revealed that many British Asian Muslim students arrived late or even missed Friday afternoon laboratory sessions.

*Initiatives:* Sessions were rescheduled for the most affected cohort to avoid conflict with Friday prayers [11]. Key taught sessions were also adjusted to avoid Eid, and mixed lab groups were introduced to encourage diverse peer learning and reduce cultural clustering [12].

*Impact:* These changes made it easier for students to practise their faith, supporting and facilitating their cultural observance. The adjustments are now embedded annually, highlighting how a relatively minor shift can yield significant positive outcomes.

### Curriculum design and delivery

*Rationale:* Our Portfolio Refresh enabled a whole-programme approach to embedding inclusivity, moving beyond isolated changes to systemic integration. Drawing on Hockings’ [13] inclusive pedagogy, responsive to student diversity, and Gay’s [14] culturally responsive pedagogy, we centred students’ cultural knowledge and experience as key to engagement. We also address the hidden curriculum [15] by making implicit elements visible and inclusive, thereby supporting equity.

*Initiatives*: Curricula were diversified through global case studies, inclusive reading lists and the integration of historically excluded scientists. Modules (e.g. those focusing on infection) embedded socio-cultural contexts to explain health inequalities and varied clinical presentations, reducing bias and avoiding stigmatisation.

*Impact*: From the LTA (Learning, Teaching and Assessment) Lead and Student Voice: *Students reported feeling represented and highlighted the positive effect of diversified and globalised content delivered*.

### Assessment and feedback

As part of the renewed portfolio, we reformed assessment to prioritise authentic learning, wellbeing and reducing anxiety. Co-created with students [16], rubrics and marking briefs together with AI support sessions enhanced clarity. Constructive alignment [17] guided the design of applied lab coursework and theory-synthesis exams, supported by scaffolded lab learning.

*Impact:* Staff reported increased confidence in inclusive practice. Students felt better presented and prepared, noting improved fairness, clarity and preparedness (Table 1).

**Table 1:** Feedback on inclusive assessment practices

Role	Task	Quote
LTA lead	Inclusion of formative assessment checkpoint	*‘*Building in regular formative checkpoints throughout our semester one skills modules has made a noticeable difference to students’ confidence and academic practice in the early stages of the program*’*.
Skills module lead	Co-creating of assessment briefs	*‘*Working with students to co-create assessment briefs has been one of the simplest ways to improve accessibility and clarity of the assessment task*’.* *‘*Co-designed rubrics incorporated student feedback. The language was simplified to clarify tasks linked to marks, enabling students to self-assess. The final version was praised by the student representative as supporting student success*’*.
Module lead	Increasing clarity of marking grids	*‘*Inviting students into conversations about marking grids has made the whole process more transparent. It has also helped us to see where the language we use is not as clear or inclusive as we think*’.*

LTA, Learning Teaching and Assessment.

### Community and sense of belonging

*Rationale:* Belonging and representation are critical for success, retention and engagement [18]. In STEM, minoritised students often lack visible role models [19], reinforcing systemic inequalities. Equity-focused dialogue and intentional spaces for marginalised voices promote critical reflection, disrupt dominant narratives and drive institutional change [20,21].

*Initiatives:* (**A**) Identity-Affirming Peer Network – Three peer-led groups: Minoritised Students, LGBTQ+ and Accessibility Support Group (non-neurotypical, neurodivergent) were co-developed with students, providing safe spaces for dialogue and advocacy and embodying participatory inclusivity [20]. These were informed by Freire’s [22] critical pedagogy and position students as co-creators of knowledge and change [20,21]. (**B**) Enhancing Visibility in STEM – An EEDI (Equality, Equity, Diversity and Inclusion) Site was launched on our Virtual Learning Environment to celebrate cultural events, showcase diverse scientists and amplify student voices. This was complemented by visual displays featuring Black and minoritised scientists including our PhD students as well as the use of social media (TikTok). (**C**) Equity-Focused Seminars – Seminars aligned with Black History Month, LGBTQ + History Month and International Women’s Day, alongside a Chemistry seminar series supported by a Royal Society of Chemistry Inclusion and Diversity Grant, were delivered to spotlight underrepresented scientists and normalise inclusive discourse.

*Impact:* Sessions inspired staff and students, broadened representation, challenged stereotypes and strengthened the School’s commitment to equity, as highlighted in (Table 2).

**Table 2:** Staff and student feedback on seminar series

Staff feedback	Student feedback
*‘*The EDI seminars have been deeply impactful on my practice. Minoritised scientists have had a seemingly safe space to discuss their studies, career, the biases they have faced and how they have responded to them*’*.	*‘*It was eye-opening to hear different perspectives on barriers I hadn’t realised were so significant. The talks highlighted how much more needs to be done to be inclusive and supportive. Clive’s session was particularly valuable in showing how mindsets can change and develop*’*.
*‘*I found the recent session both inspiring and relevant. It encouraged me to reflect on how I can act positively and reaffirmed that I can add value while embracing my identity as a Muslim and a person of Black origin. The discussion on personal branding was particularly impactful, highlighting the importance of showcasing skills and contributions without compromising authenticity*’*. *‘*The talk left me confident that, despite being different, I can make a positive impact and remain resilient while being proud of my identity*’*. *‘*Thank you for organising these sessions, they truly matter to those of us from minority backgrounds*’.*	*‘*I found the talks inspiring, but also grounded in the realities of the challenges that still exist for individuals from minority backgrounds, which made them very impactful*’.*
*‘*The EDI seminars have been deeply impactful on my practice. Minoritised scientists have had a seemingly safe space to discuss their studies, career, the biases they have faced and how they have responded to them*’.*	*‘*It has helped me confront my own bias, make sure I engage with EDI and ensure I always advocate for others*’.*
*‘*The breadth of voices giving a platform within these seminars has allowed me to reflect on the importance of representation and gain a greater understanding of how to be an ally to students and colleagues. This has then given me the confidence to talk more openly about minoritised scientists and groups as well as be a vocal advocate for EDI in my teaching*’.*	*‘*I wanted to hear directly from a senior LGBTQ + scientist because role models and personal stories are rare in science*’.*
	*‘*It was really useful to hear other perspectives on issues and barriers that people face that I didn't realise were such significant issues. The talks were really useful to help me understand that a lot more work is needed to be done to be more inclusive and supportive. Clive’s talk was also really useful about understanding different mindsets and mentalities that we have and understanding that we can all learn to change and develop them*’*.

### Pathways to success

#### Outreach and transition

*Rationale* **:** Students’ sense of belonging starts pre-enrolment [23], and the Hubbard and Gawthorpe Framework places outreach within Pathways to Success, recognising its role in building early confidence [24].

*Initiatives* ***:*** Outreach and recruitment were designed to engage diverse prospective students through inclusive events and transparent communication. Compared to 2022/2023, engagement rose significantly; activities delivered increased to 58 (81% growth), student participation reached 1285 (32% increase), and teacher/advisor involvement grew to 103 (80% increase). Over three years, over 1000 students and 100 teachers participated in activities led by diverse staff and student ambassadors. Pre-arrival materials and identity-affirming inclusion activities were expanded to foster early connection and visibility.

*Impact* **:** Increased student applications, together with positive feedback and a stronger early sense of belonging, were reported (Table 3).

**Table 3:** Student reflections on inclusive open days and transitions

Courses	Student quotes
Biochemistry student	*‘*It’s like they really try to make it as easy as possible for settling in*’.*
Biochemistry student	*‘*When you came to the Open Day, they made you feel you deserved to be here*’.*
Biomedical science student	*‘*They cared about you as well, like I got a postcard that was handwritten and sent to me, which I thought was really sweet. I could actually get them on the phone because I went through this massive dilemma where I'd worried, I'd applied to the wrong course, and I could actually get them on the phone*’.*
Biomedical science student	*‘*Receiving a handwritten postcard made me feel that I was a person and they actually wanted me at the university*’.*

#### Student support: supporting mental health and well-being

*Rationale:* Mental health is foundational to student success, and supporting well-being is a key priority aligned with the University Mental Health Charter [25].

*Initiative*: Hybrid sessions were delivered in collaboration with Student Support and Health Services, and a central digital resource hub was created.

*Impact:* Staff reported that the sessions were informative and helped them direct students effectively. Students said that the sessions gave them confidence to seek advice. The Skills Module Lead reported that, *based on this, early signposting was introduced in the skills module, resulting in a record number of proactive disclosures and ensuring students were better supported*.

## A call to action

This article calls for a renewed commitment to equity in HE. Closing awarding gaps requires collective, sustained effort from the entire academic community, with every student, staff and senior leaders, all contributing to an inclusive environment where all students can thrive. The expanded examples (Table 4), now reaching beyond the mini case studies, illustrate how inclusive practices can be mapped and embedded across each stage of the student journey, highlighting that meaningful change depends on institution-wide collaboration and shared responsibility.

**Table 4:** Roles and responsibilities across the student journey

Theme	Programme lead	Course lead	Module lead	Teaching academics	Student demonstrators
**Outreach**	Set strategy, build external partnerships	Represent the course at events	Support the delivery of outreach content	Participate in open days and school visits	Share experiences, engage in taster/open days
**Transition and induction**	Lead and evaluate induction strategy	Design and co-ordinate course-level induction	Support module-level induction planning	Participate in induction activities, welcome students	Lead peer tours, offer informal guidance for new students
**Curriculum content**	Align curriculum with institutional, accreditation body and strategic goals	Ensure coherence across modules	Design, update and review module content	Develop and deliver inclusive and engaging materials	Provide feedback and suggest improvements
**Assessment**	Lead assessment strategy and ensure alignment with learning outcomes	Maintain balance and integrity of assessments across the course	Design fair and inclusive assessments	Conduct marking, moderation and provide timely feedback	Contribute feedback, support co-creation with students
**Feedback**	Monitor feedback practices and impact	Ensure consistency and quality of feedback	Review and improve feedback mechanisms	Provide timely, constructive and actionable feedback	Offer informal peer feedback and support
**Employability skills**	Embed employability and transferable skills across the programme	Organise placements, employer and career talks	Integrate employability themes into modules	Embed real-world context and skill development in teaching	Share work experiences, mentor peers, promote career awareness
**Sense of belonging**	Promote inclusive identity and community	Facilitate cohort-building and inclusive activities	Encourage inclusive practices within modules	Foster supportive and inclusive learning environments	Be relatable, create informal peer support networks
**Student voice**	Use feedback for strategic decisions and improvements	Liaise with reps, act on course-level feedback	Monitor module-level feedback and act on it	Invite feedback, co-create learning	Act as feedback conduit, advocate for peers and raise concerns
**Invited speakers**	Build relationships with industry leaders and alumni	Co-ordinate logistics, ensure alignment with course aims	Recommend and invite speakers to enhance module content	Host and engage speakers during sessions, facilitate discussion	Introduce speakers, share takeaways with peers

## Conclusion

Closing the DAG requires systemic, institution-wide change. At SHU, our approach, grounded in culturally responsive pedagogy, student voice and structural reform, demonstrates that early belonging, visible representation and strategic equity planning can drive meaningful progress. While momentum is growing, sustained impact requires continued staff engagement and long-term evaluation. Our next step is to translate these initiatives into measurable reductions in the DAG. Ultimately, closing the DAG opens doors to equity, belonging and academic success, reshaping HE so all students can thrive.

## Open Access

Open access for this article is provided via our Subscribe to Open programme, made possible through the support of our subscribing institutions. Learn more here: https://portlandpress.com/pages/open_access_options_and_prices#conditional

## Abbreviations

DAG: degree awarding gap
EDI: equality, diversity and inclusion
SHU: Sheffield Hallam University

## References

[1] Mountford-Zimdars A. Sabri D. Moore J. Sanders J. Jones S. and Higham L 2015 Causes of Differences in Student Outcomes London; HEFCE

[2] National Union of Students 2011 Race for Equality: A Report on the Experiences of Black Students in Further and Higher Education Race for equality

[3] Alexander C 2015 Aiming Higher: Race, Inequality and Diversity in the Academy London: Runnymede Trust

[4] Office for Students 2024 KPM 7: Degree attainment by ethnicity – 2022–23 https://www.officeforstudents.org.uk/about/how-we-are-run/key-performance-measures/kpm-7-degree-attainment-by-ethnicity/

[5] Ugiagbe-Green I. and Ernsting F 2022 The wicked problem of B(A)ME degree award gaps and systemic racism in our universities Front. Sociol. 7 971923 10.3389/fsoc.2022.971923 36267119 PMC9576997

[6] Li P.-Hsin 2024 Student experience in HEIs: What does the evidence say about the ethnicity awarding gap? The Diversity of Student Experience Project The Centre for Teaching and Learning. University of Oxford https://www.ctl.ox.ac.uk/sitefiles/dse-lit-ethnicity-april-24.pdf

[7] Hubbard K 2021 Using data-driven approaches to address systematic awarding gaps In Doing Equity and Diversity for Success in Higher Education: Redressing Structural Inequalities in the Academy ( Thomas D. and Arday J. eds pp 215 226 Springer

[8] Hubbard K. and Gawthorpe P 2024 Inclusive Higher Education Framework. National Teaching Repository Educational resource https://www.inclusiveeducationframework.info/

[9] Advance H.E 2020 A framework for supporting governing body effectiveness reviews in higher education https://www.advance-he.ac.uk/knowledge-hub/framework-supporting-governing-body-effectiveness-reviews-higher-education

[10] Blell M. Liu S.S. and Verma A 2023 Working in unprecedented times: Intersectionality and women of color in UK higher education in and beyond the pandemic Gender. Work & Organization 30 353 372 10.1111/gwao.12907

[11] Islam M. Lowe T. and Jones G 2018 A ‘satisfied settling’? Investigating a sense of belonging for Muslim students in a UK small-medium Higher Education Institution Student Engagement in Higher Education Journal 2 79 104

[12] Liang Y. and Schartner A 2022 Culturally mixed group work and the development of students’ intercultural competence Journal of Studies in International Education 26 44 60 10.1177/1028315320963507

[13] Hockings C 2010 Inclusive Learning and Teaching in Higher Education: A Synthesis of Research Higher Education Academy

[14] Gay G 2018 Culturally Responsive Teaching: Theory, Research, and Practice. Teachers College Press 3rd ed ed New York Teachers College Press)

[15] Koutsouris G. Mountford-Zimdars A. and Dingwall K 2021 The ‘ideal’ higher education student: understanding the hidden curriculum to enable institutional change Research in Post-Compulsory Education 26 131 147 10.1080/13596748.2021.1909921

[16] Bovill C 2020 Co-creation in learning and teaching: the case for a whole-class approach in higher education High. Educ. 79 1023 1037 10.1007/s10734-019-00453-w

[17] Biggs J. and Tang C 2003 Teaching for quality learning Buckingham: Society for Research into Higher Education and Open University Press https://cetl.ppu.edu/sites/default/files/publications/-John_Biggs_and_Catherine_Tang-_Teaching_for_Quali-BookFiorg-.pdf

[18] Palmer E 2024 Fostering a sense of belonging In Supporting the Student Journey into Higher Education 2024 Mar pp 35 41 Routledge

[19] Weaver G.R. Treviño L.K. and Agle B 2005 Somebody I look up to:”: Ethical role models in organizations Organ. Dyn. 34 313 330 10.1016/j.orgdyn.2005.08.001

[20] Burkholder C. Thorpe A. and Swell P 2022 Facilitating gender-affirming participatory visual research in embodied and online spaces Visual Studies 37 33 46 10.1080/1472586X.2021.1982650

[21] O’Connor R. Barraclough L. Gleadall S. and Walker L 2025 Institutional reverse mentoring: Bridging the student/leadership gap Br. Educ. Res. J. 51 344 368 10.1002/berj.4078

[22] Freire P 1970 Pedagogy of the Oppressed, New York Herder & Herder

[23] Thomas L 2012 Building student engagement and belonging in Higher Education at a time of change Paul Hamlyn Foundation 100 1 02

[24] Walton G.M. and Cohen GL 2007 A question of belonging: race, social fit, and achievement J. Pers. Soc. Psychol. 92 82 96 82 10.1037/0022-3514.92.1.82 17201544

[25] Hughes G. and Spanner L 2024 The University Mental Health Charter 2nd ed Student Minds

